# End-of life thoughts in the ICU: results of a survey

**DOI:** 10.1186/cc12467

**Published:** 2013-03-19

**Authors:** A Yaguchi, M Namiki, N Saito, R Nagai, M Takeda, T Harada, R Moroi

**Affiliations:** 1 Tokyo Women's Medical University, Tokyo, Japan

## Introduction

The decision of terminal care in the ICU is a very tough issue because the law, ethics, traditions and futility should be concerned involving the family's will. Especially, stopping or withdrawing therapy is a quite difficult operation in Japan because of legal issues. Our hypothesis is that some difference exists in thoughts between physicians and nurses for terminal patients in the ICU. The aim of this study is to know their real thoughts.

## Methods

A questionnaire survey was performed on physicians and nurses in our medico-surgical ICU. The questionnaire consists of 11 questions with five optional answers related to the thoughts of participants about treatment of hopeless or brain death patients. Concretely, the questions were; whether to withhold therapy or not, whether to accept to withdraw therapy or not and with family's will, whether to accept to immediately stop therapy and with family's will, whether to positively or not donate organs from a brain death patient, necessity of ICU care for brain death patients, and feeling guilty and stress for stopping or withdrawing therapy. The optional answer has five gradations from 'Yes' to 'No' for all questions. The participants were asked to answer the questionnaire by expressing themselves without regarding legal issues or the consensus. It was guaranteed to be anonymous for them in the data analysis. The answers were compared between physicians and nurses. The Mann-Whitney *U *test was used for statistical analysis. *P *0.05 was considered statistically significant.

## Results

There were in total 52 participants (response rate 98.1%) with 20 physicians and 32 nurses. Withdrawing therapy was significantly accepted in nurses than in physicians (83% vs. 55%, *P *= 0.039), when the family well understood. Withholding therapy should not be operated for brain death patients for physicians (65%), while it seemed a difficult judgement for nurses (23%, *P *= 0.021). ICU care for brain death patients is less necessary for physicians than nurses (80% vs. 53%, *P *= 0.016). There were no significant differences in other questions between physician and nurses such as feeling guilty or stress for stopping or withdrawing therapy.

## Conclusion

Some of end-of-life thoughts in the ICU showed differences between physicians and nurses.

